# Human placenta-derived mesenchymal stem cells stimulate ovarian function via miR-145 and bone morphogenetic protein signaling in aged rats

**DOI:** 10.1186/s13287-020-01988-x

**Published:** 2020-11-05

**Authors:** Kyeoung-Hwa Kim, Eun-Young Kim, Gi Jin Kim, Jung-Jae Ko, Kwang Yul Cha, Mi Kyung Koong, Kyung-Ah Lee

**Affiliations:** 1 Department of Biomedical Science, Institute of Reproductive Medicine, College of Life Science, CHA University, Pangyo-Ro 335, Bundang-gu, Seongnam-si, Gyeonggi-do 13488 South Korea; 2 CHA Stem Cell Institute, CHA University, Pangyo-Ro 335, Bundang-gu, Seongnam-si, Gyeonggi-do 13488 South Korea; 3 CHA Fertility Center Seoul Station, CHA University School of Medicine, 416, Hangang-daero, Jung-gu, Seoul, 04637 South Korea

**Keywords:** Aging, Stem cell therapy, Hormone biosynthesis, miR-145, Primordial follicle activation, Follicular development

## Abstract

**Background:**

Aging has detrimental effects on the ovary, such as a progressive reduction in fertility and decreased hormone production, that greatly reduce the quality of life of women. Thus, the current study was undertaken to investigate whether human placenta-derived mesenchymal stem cell (hPD-MSC) treatment can restore the decreases in folliculogenesis and ovarian function that occur with aging.

**Methods:**

Acclimatized 52-week-old female SD rats were randomly divided into four groups: single hPD-MSC (5 × 10^5^) therapy, multiple (three times, 10-day intervals) hPD-MSC therapy, control (PBS), and non-treated groups. hPD-MSC therapy was conducted by tail vein injection into aged rats. The rats were sacrificed 1, 2, 3, and 5 weeks after the last injection. hPD-MSC tracking and follicle numbers were histologically confirmed*.* The serum levels of sex hormones and circulating miRNAs were detected by ELISA and qRT-PCR, respectively. TGF-β superfamily proteins and SMAD proteins in the ovary were detected by Western blot analysis.

**Results:**

We observed that multiple transplantations of hPD-MSCs more effectively promoted primordial follicle activation and ovarian hormone (E_2_ and AMH) production than a single injection. After hPD-MSC therapy, the levels of miR-21-5p, miR-132-3p, and miR-212-3p, miRNAs associated with the ovarian reserve, were increased in the serum. Moreover, miRNAs (miR-16-5p, miR-34a-5p, and miR-191-5p) with known adverse effects on folliculogenesis were markedly suppressed. Importantly, the level of miR-145-5p was reduced after single- or multiple-injection hPD-MSC therapy, and we confirmed that miR-145-5p targets *Bmpr2* but not *Tgfbr2*. Interestingly, downregulation of miR-145-5p led to an increase in BMPR2, and activation of SMAD signaling concurrently increased primordial follicle development and the number of primary and antral follicles.

**Conclusions:**

Our study verified that multiple intravenous injections of hPD-MSCs led to improved ovarian function via miR-145-5p and BMP-SMAD signaling and proposed the future therapeutic potential of hPD-MSCs to promote ovarian function in women at advanced age to improve their quality of life during climacterium.

## Background

The ovaries are the main female reproductive organs that sustain reproductive system health and safeguard the quality of life of women through hormone biosynthesis. However, the aging of the ovaries occurs faster than that of other reproductive organs, such as the uterus and pituitary gland [1]. Ovarian aging is accompanied by a significant decline in the number of primordial follicles, an ovarian reservoir, and an increased number of low-quality oocytes, which contribute to the gradual decline in fertility and thus the increase in the incidence of natural sterility caused by menopause [2, 3]. Additionally, menopause is the final step in the process referred to as ovarian aging [3]. Aging-associated menopause leads to systemic complications such as menopausal symptoms, metabolic syndrome, dementia, osteoporosis, and heart disease [4, 5]. Female reproductive aging is an increasing public health issue in modern society in which women pursue a career long before marriage at more than 30 years of age.

A primordial follicle consists of an immature oocyte arrested at the diplotene stage of meiosis I surrounded by several flattened pregranulosa cells [6]. At birth, the fetal ovary usually contains approximately 300,000 to 400,000 primordial follicles, and this number declines with age [7, 8]. At the beginning of puberty, quiescent primordial follicles develop into primary follicles under the control of AKT and MTOR signaling induced by stimulation factors [9]. These factors include kit ligand, VEGF, BMP4, BMP7, LIF, FGF2 (known as basic FGF), and others [10–15]. However, most follicles undergo apoptosis, a process known as atresia, and only 300 to 400 follicles are selected for ovulation during the reproductive lifetime of an adult female [16]. Therefore, the pool of primordial follicles in the ovarian reserve is the major determinant of the reproductive period, healthy ovarian lifespan, and thus the fertility and reproductive health of an individual [17].

Circulating microRNAs (miRNAs) are small noncoding RNA molecules that negatively regulate gene expression through mRNA cleavage, translational repression, or mRNA degradation [18]. It has also been shown that circulating miRNAs in serum or plasma can modulate gene expression during aging processes and can affect aging phenotypes [19]. Thus, circulating miRNAs have recently emerged as important regulators of aging and frailty in elderly individuals. In addition, several studies have identified circulating miRNA populations specifically associated with ovarian function, such as follicle development, ovulation, granulosa cell apoptosis, and steroidogenesis [20, 21]. Therefore, ovarian function-related and/or ovarian-specific circulating miRNAs detected in the serum can be used as early diagnostic and prognostic monitoring markers in ovarian dysfunction with aging.

In previous studies, human placenta-derived mesenchymal stem cell (hPD-MSC) transplantation was shown to have positive effects on degenerative diseases, such as antifibrosis, anti-inflammation, and antiapoptosis effects [22–24]. Moreover, transplantation of hPD-MSCs had therapeutic effects on young ovariectomized (Ovx) rats by increasing the levels of E_2_ and folliculogenesis-related gene expression [25]. However, whether hPD-MSC therapy can restore ovarian follicular development and the other functions of naturally aged ovaries is still unknown. Thus, the aims of this study were to determine whether hPD-MSC therapy could counteract age-related ovarian dysfunctions and decrease ovarian reserve in advanced-age rat models and to evaluate the efficacy of single-injection hPD-MSC therapy and multiple-injection hPD-MSC therapy on age-associated physiological characteristics, including ovarian functions, in old female rats.

## Methods

### Reagents

Chemicals and reagents were obtained from Sigma-Aldrich (MO, USA) unless otherwise noted.

### Experimental design

All experiments involving animals were approved by the Institutional Animal Care and Use Committee (IACUC 190163) of the CHA Laboratory Animal Research Center. Sprague-Dawley (SD) rats were provided by Janvier Labs (Le Genest-Saint-Isle, France) and maintained at the breeding facility at the CHA Bio Complex of CHA University. SD rats (52–54 weeks of age) were used for all experiments. These animals were randomly divided into single- and multiple-injection hPD-MSC therapy groups (*n* = 24 each group), a control group, and an untreated group. For single-injection hPD-MSC therapy, 5 × 10^5^ hPD-MSCs were suspended in 0.2 ml of phosphate-buffered saline (PBS) and injected into rats via the tail vein. For multiple-injection hPD-MSC therapy, a series of PKH67-labeled hPD-MSCs were injected three times at 10-day intervals or 4-week intervals. The rats from the control group were only injected with an equal volume of PBS. The rats were sacrificed 1, 2, 3, and 5 weeks following the transplantation of hPD-MSCs, and the organs and blood samples were collected and immediately frozen. Ovaries were fixed with 4% paraformaldehyde or optimal cutting temperature (OCT) compound (Leica Biosystems, IL, USA) for further processing. Serum was collected by centrifugation and stored at − 80 °C for hormone assays.

### Culture of hPD-MSCs

hPD-MSCs were kindly provided by Dr. Gi Jin Kim. hPD-MSCs were isolated and characterized as described previously [22, 25]. Placentas (38 ± 2 gestational weeks) were collected from women who were free of any medical, obstetrical, or surgical complications. All participants provided written informed consent prior to placenta collection. hPD-MSCs were isolated from human placental chorionic plates and approved by the Institutional Review Board of CHA General Hospital, Seoul, Korea (IRB 07-18). Briefly, the fetal membrane was removed from the chorionic plate of each placenta, and cells were treated with 0.5% collagenase IV (Sigma-Aldrich) for 30 min at 37 °C. The harvested cells were plated (2 × 10^5^ cells/cm^2^) with α-MEM supplemented with 10% fetal bovine serum (FBS; Corning, NY, USA), 1% penicillin-streptomycin (Life Technologies, USA), 1 mg/ml heparin (Sigma-Aldrich), and 100 μg/ml FGF4 (PeproTech, NJ, USA). The cells were maintained in a humidified atmosphere containing 5% CO_2_ and grown until 80–90% confluence.

### Labeling and tracking of hPD-MSCs

To track and locate the transplanted hPD-MSCs in the ovarian tissues, the cells were prelabeled with PKH67 Green Fluorescent Cell Linker kits (Sigma-Aldrich) according to the manufacturer’s instructions. Briefly, a total of 2 × 10^7^ hPD-MSCs were washed and gently resuspended in 1 ml of Diluent C. In parallel, 4 μl of PKH67 dye was added to 1 ml of Diluent C (4 × 10^−6^ M) and incubated with the hPD-MSC solution for 5 min. To bind excess dye, the same volume of 1% BSA was added. The labeled hPD-MSCs were washed and observed by fluorescence microscopy.

PKH67-labeled hPD-MSCs were transplanted into rats via the tail vein. Ovaries were fixed with OCT compound and made into fresh sections (12 μm thick). After fixation with 4% paraformaldehyde for 20 min, the sections were washed and incubated with 2-(4-amidinophenyl)-6-indolecarbamidine dihydrochloride (DAPI; Sigma-Aldrich) at room temperature for 10 min. The sections were then imaged under a laser scanning confocal microscope (LSCM; Leica, Wetzlar, Germany).

### Genome extraction

Rat genomic DNA was isolated from the ovary, liver, and lung after hPD-MSC therapy as described previously [26]. After RNA extraction with 500 μl of TRIzol (Invitrogen, CA, USA), we added 250 μl of back extraction buffer (BEB; 4 M guanidine thiocyanate; 50 mM sodium citrate; 1 M Tris, pH 8.0) and allowed the interphase-organic phase mixtures to sit at room temperature for 10 min. The samples were then centrifuged at 13,000 rpm for 15 min at 4 °C. The upper phase was removed, an equal volume of 100% isopropanol was added, and the samples were incubated overnight at − 80 °C. After incubation, the samples were centrifuged again at 13,000 rpm for 15 min at 4 °C. The supernatant was removed, and the pellets were washed 3 times with 70% ethanol. The samples were eluted in a final volume of 30 μl of Tris-EDTA (10 mM Tris; 0.1 mM EDTA, pH 8.0).

### Human Alu sequence and RT-PCR

We synthesized the primers as described previously by Walker et al. [27]. The primer sequences were as follows: forward primer (*AluYb8*_F; position 48-69), 5′-CGAGGCGGGTGGATCATGAGGT-3′; reverse primer (*AluYb8*_R; position 273-254), 5′-TCTGTCGCCCAGGCCGGACT-3′.

The extracted genomic DNA was quantified using a NanoDrop UV spectrophotometer (Thermo Fisher Scientific, MA, USA). RT-PCR was performed with *AluYb8* primers and 100 ng of genomic DNA from the various organs after single-injection hPD-MSC therapy. RT-PCR products were separated by electrophoresis on a 1.5% agarose gel and analyzed using a Gel Doc™ EZ Imager (Bio-Rad, CA, USA).

### Ovarian morphology and follicle counting

To analyze ovarian morphology and follicle counts, six ovaries from each group were collected at the indicated time points (1, 2, 3, and 5 weeks after single- or multiple-injection hPD-MSC therapy) and fixed with 4% paraformaldehyde for 48 h at 4 °C. The ovaries were dehydrated, embedded in paraffin, and serially cut into 7-μm sections. After hematoxylin and eosin (H&E) staining, sections were observed and imaged by an optical microscope (Nikon Corporation, Tokyo, Japan). Each follicle stage, namely primordial, primary, secondary, preantral, and antral follicle stages, was counted in every tenth section through the ovary.

### Circulating miRNA extraction

Circulating miRNAs were extracted from 200 μl of serum using an miRNeasy serum/plasma kit (Qiagen, Hilden, Germany) according to the manufacturers’ instructions. Briefly, serum samples were lysed with 1 ml of Qiazol and spiked with 3.5 μl of synthetic cel-miR-39 mimic (1.6 × 10^8^ copies/μl; Qiagen) to test extraction efficiency. Chloroform (200 μl) was used for phase separation at 4 °C and 10,000 rpm. The aqueous phase (600 μl) was added to 900 μl of 100% ethanol (Merck Millipore, MA, USA) and subsequently transferred into an RNeasy MiniElute spin column. These columns were washed with RWT buffer, RPE buffer, and 80% ethanol (Merck Millipore). All samples were eluted in 14 μl of RNase-free water.

### Quantitative real-time RT-PCR (qRT-PCR)

For reverse transcription (HB_I RT Reaction kit; Heimbiotek, Pangyo, Korea), 11 μl of the eluted miRNAs was mixed with 2 μl of miR enzyme mix, 2 μl of miR multi buffer, and 5 μl of nucleic mix I. The samples were incubated for 60 min at 37 °C and for 5 min at 95 °C.

To measure the amounts of circulating miRNAs in serum after hPD-MSC therapy, quantitative real-time RT-PCR analysis was performed using a CFX96 Touch™ Real-Time PCR Detection System (Bio-Rad). The primers used to amplify the selected miRNAs were purchased from Heimbiotek (Nucleic Mix II). The HB miR multi assay kit system I (Heimbiotek) was used for monitoring amplification, and the results were evaluated using CFX Maestro software (Bio-Rad). Melting curves were used to identify nonspecific amplification products. The expression of each miRNA was normalized to the expression of cel-miR-39. The relative target miRNA expression was calculated using the comparative C_T_ method.

### Western blotting

To confirm protein expression, Western blotting was performed as previously described [28]. Briefly, a protein extract was separated using 10% or 12% SDS-PAGE and transferred onto a PVDF membrane (Amersham Biosciences, Piscataway, NJ). After blocking, the membrane was incubated with antibodies against BMPR2 (1:1000; ab96826, Abcam, Cambridge, MA), TGFBR2 (1:1000; #79424, Cell Signaling Technology, Danvers, MA), ACVR1B (1:1000; ab109300, Abcam), ACVR2A (1:1000; ab96793, Abcam), SMAD1 (1:1000; #9743, Cell Signaling Technology), phospho-SMAD1/5 (p-SMAD1/5; 1:1000; #9516, Cell Signaling Technology), SMAD3 (1:1000; #9513, Cell Signaling Technology), phospho-SMAD3 (p-SMAD3; 1:1000; #9520, Cell Signaling Technology), and GAPDH (1:2000; sc-47,724, Santa Cruz Biotechnology, Dallas, TX), followed by incubation with HRP-conjugated anti-rabbit IgG (1:5000; #7074, Cell Signaling Technology, Danvers, MA, USA) or anti-mouse IgG (1:2000; A2554). Bound antibodies were detected using an enhanced chemiluminescence detection system (Amersham Biosciences) according to the manufacturer’s instructions.

### Levels of E_2_ and AMH in serum

One sample of 2 ml of blood was collected from each rat. The blood samples were centrifuged, and the serum was separated into two parts: one part was used for AMH analysis, and one part was used for E_2_ analysis. The serum levels of AMH and E_2_ at the indicated time points (1, 2, 3, and 5 weeks after single- or multiple-injection hPD-MSC therapy) were measured using the Elecsys® AMH immunoassay (Roche Diagnostics GmbH, Mannheim, Germany) and Elecsys® Estradiol III (Roche Diagnostics GmbH), respectively. All serum markers (AMH and E_2_) were determined in a single measurement on the e601 module of the fully automated Cobas 6000 system (Roche Diagnostics GmbH). Assays were performed according to the manufacturer’s instructions.

### Statistical analysis

Data are expressed as the mean ± SEM. Statistical analyses were performed using Student’s *t* test for paired samples (non-treated group vs. single-injection hPD-MSC therapy group; control group vs. multiple-injection hPD-MSC therapy group), and *p* < 0.05 was considered statistically significant.

## Results

### hPD-MSCs were detected in ovaries after cell therapy

**Fig. 1:**
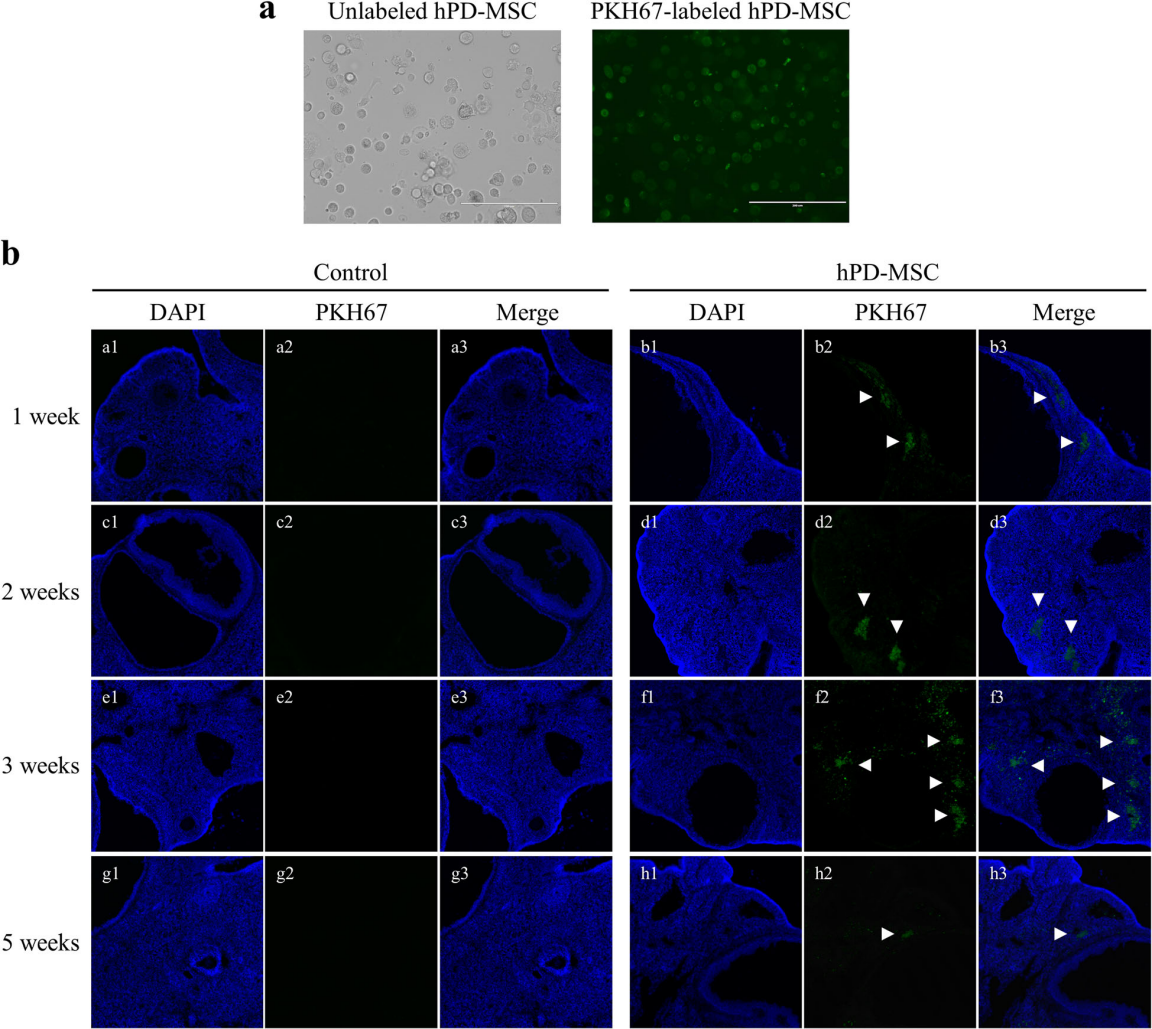
PKH67-labeled hPD-MSCs were detected in the interstitium and near the follicular region in the ovaries after therapy. **a** PKH67-labeled hPD-MSCs showed green fluorescence. The scale bars indicate 200 μm. **b** PKH67-labeled hPD-MSCs (green) were observed in ovaries 1 week (b1–b3), 2 weeks (d1–d3), 3 weeks (f1–f3), and 5 weeks (h1–h3) after multiple-injection hPD-MSC therapy. Note the appearance of numerous PKH67-labeled hPD-MSCs in the interstitium of ovaries and some PKH67-labeled hPD-MSCs in the ovaries near the follicular region (white arrowheads). DNA was counterstained with DAPI (blue). Control, PBS-injected group; hPD-MSCs, PKH67-labeled multiple-injection hPD-MSC group. The scale bars indicate 200 μm

To track and locate hPD-MSCs in the ovary, the cells were prelabeled with PKH67 before injection. The staining of hPD-MSCs with PKH67 revealed consistent, clear, and uniform distribution of the labeled cell membrane as observed using an inverted microscope (Fig. 1a). The location and fate of injected PKH67-labeled hPD-MSCs in ovarian tissues were tracked at 1 week, 2 weeks, 3 weeks, and 5 weeks after tail vein injection (Fig. 1b). The results show that PKH67-labeled hPD-MSCs were located in the interstitium and surrounding the follicles (white arrowheads) of ovaries after multiple-injection therapy. We found that PKH67-labeled hPD-MSCs were observed in the therapy groups, starting on the first week after multiple cell injections via the tail vein (Fig. 1b (b1–3)). In addition, a green fluorescent signal could still be observed in ovaries at 5 weeks after the multiple-injection therapy (Fig. 1b (h1–3)). However, the green fluorescent signal gradually decreased with time (Fig. 1b). On the other hand, human DNA (*AluYb8*) sequences were found after single-injection therapy by tail vein injection. We detected the PCR products of *AluYb8* sequences in ovaries 2–3 weeks after single-injection therapy (Additional file 1: Figure S1). These results indicate that hPD-MSCs were detected faster in ovaries and showed long-term maintenance after multiple hPD-MSC injections than after a single injection.

### hPD-MSC therapy induced follicular development and steroid production

**Fig. 2:**
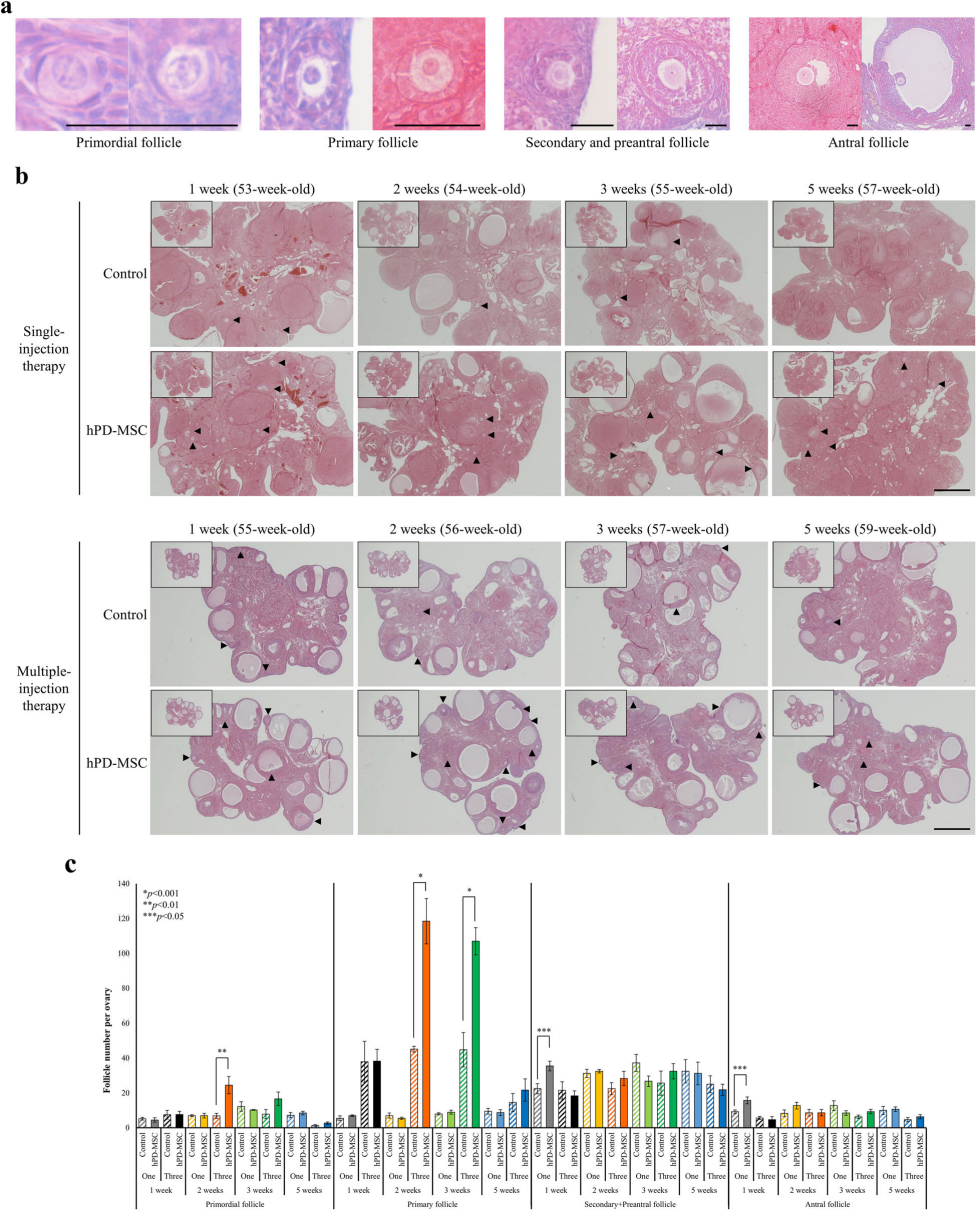
hPD-MSC therapy attenuates ovarian dysfunction by inducing follicular development. **a** Classification of the ovarian follicular stages used for analysis. The scale bars indicate 50 μm. **b** Ovarian structure after hPD-MSC therapy. Six rats in each group were randomly selected at the indicated time points following hPD-MSC injection. By histological analyses, serially sectioned whole ovaries were stained with H&E, and follicles at different stages were counted in the control and hPD-MSC therapy groups. Black arrowheads indicate ovarian follicles. Scale bars indicate 1 mm. **c** After single- or multiple-injection therapy, the numbers of follicles at various stages were counted and compared with the numbers in the control group. hPD-MSC therapy resulted in a significant increase in the number of developmental follicles. The results are presented as the mean ± SEM, and statistical *p* values were calculated; *p < 0.001, **p < 0.01, and ***p < 0.05. One, single-injection hPD-MSC therapy; Three, multiple-injection hPD-MSC therapy

To explore the effects of hPD-MSC therapy on the decreased ovarian reserve induced by aging, histological analysis was performed. For this study, follicles have been classified according to their morphological characteristics acquired during follicular development, including the diameter, oocyte diameter, and the granulosa cell number and morphology. A primordial follicle was defined as a small oocyte surrounded by flattened pregranulosa cells; a primary follicle was defined as having a larger oocyte surrounded by one layer of cuboidal and proliferative granulosa cells; secondary and preantral follicles were defined as follicles with a larger diameter surrounded by two or more layers of granulosa cells with no antrum; and an antral follicle was characterized by granulosa cells with more than one small antral cavity or with one large antrum (Fig. 2a). Histological studies of the ovaries found no significant difference in the number of primordial and primary follicles after single-injection therapy (Fig. 2b, c). However, there were significant increases in the numbers of secondary, preantral, and antral follicles at 1 week after single-injection hPD-MSC therapy compared with the control (Fig. 2b, c; gray striped bar vs. gray bar). These changes were improved by cell transplantation in the single-injection therapy group. Surprisingly, the number of primary follicles substantially increased at 2 weeks and 3 weeks after tail vein injection in the multiple-injection therapy group compared with the control group (Fig. 2b, c; 2 weeks, orange striped bar vs. orange bar; 3 weeks, green striped bar vs. green bar). These results appear to represent the strong positive effects of the multiple-injection therapy on early folliculogenesis. We observed a more than 2-fold increase in the number of primary follicles following multiple-injection hPD-MSC therapy compared to that following single-injection therapy (Fig. 2c). Taken together, these results demonstrate that both the single- and multiple-injection therapies can rescue the ovarian reserve and follicle development and that multiple-injection hPD-MSC therapy was more effective for primordial follicle activation and subsequent follicular development than a single injection.

Unexpectedly, we found that the number of primordial follicles was significantly increased 2 weeks after multiple-injection therapy compared with the control group (Fig. 2c; primordial follicle, 2 weeks, orange striped bar vs. orange bar). We thought that this increased number of primordial follicles in ovaries after hPD-MSC injection was a coincidence rather than an effect of the multiple-injection therapy because the total number of primordial follicles is fixed at birth, and a rat with a larger initial ovarian reservoir was assigned to that group despite randomly dividing the rats.

**Fig. 3:**
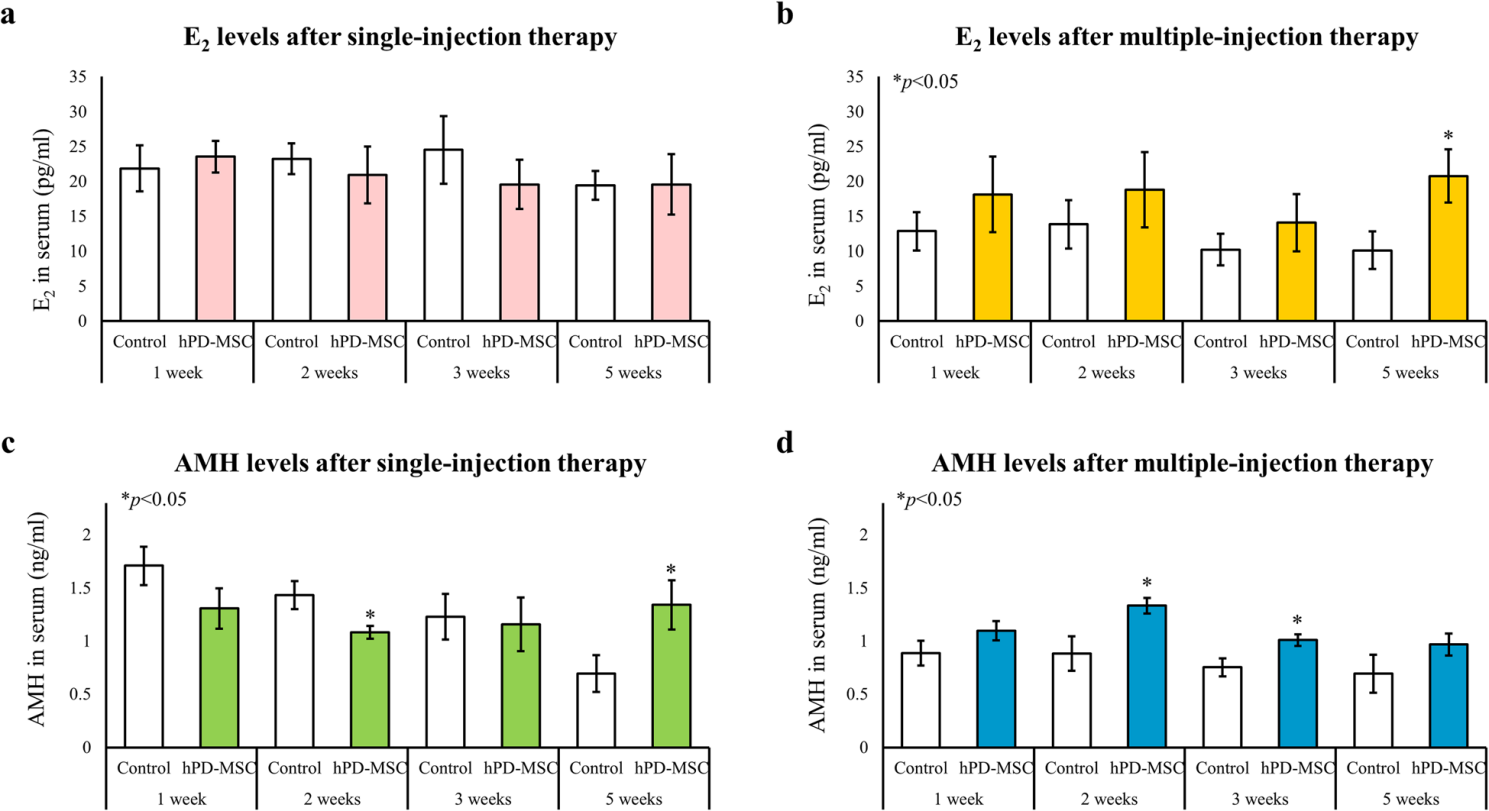
hPD-MSC therapy improves serum levels of E_2_ and AMH in aged rats. Levels of E_2_ (**a**, **b**) and AMH (**c**, **d**) as determined by ELISA at various time points after single- or multiple-injection hPD-MSC therapy. Data are presented as the mean ± SEM. The asterisk represents statistical significance at *p* < 0.05

To determine whether single-injection hPD-MSC therapy or multiple-injection hPD-MSC therapies could improve ovarian dysfunction with aging, the levels of E_2_ and AMH in serum were measured. We observed that E_2_ levels were not significantly different between the control and single-injection therapy groups (Fig. 3a); however, we found that E_2_ levels were significantly increased at 5 weeks after multiple-injection hPD-MSC therapy (Fig. 3b). As shown in Fig. 3c, the levels of AMH in the control group were gradually reduced over time. The levels of AMH in the cell therapy group slightly decreased over weeks 1–2 but increased over weeks 3–5 after therapy, showing a sustained profile (Fig. 3c). Moreover, the levels of AMH were markedly increased in the single-injection cell therapy groups at 5 weeks compared to the control group (Fig. 3c). Because the first sampling time point for the multiple-injection therapy group was 20 days (approximately 3 weeks) after than that for the single-injection therapy group, the level of AMH was relatively low in the multiple-injection therapy groups (Fig. 3d). Furthermore, we observed that AMH levels were gradually reduced in the control group over time, while after multiple-injection hPD-MSC therapy, we found that AMH levels were significantly increased (Fig. 3d). These results imply that hPD-MSC therapy may enhance AMH secretion by promoting granulosa cell proliferation and regulating follicle development. Interestingly, AMH levels increased at a faster rate in serum in the multiple-injection hPD-MSC therapy group than in the single-injection therapy group, with the multiple-injection hPD-MSC therapy group showing higher AMH levels 2–3 weeks after injection (Fig. 3c, d). Thus, the multiple-injection therapy was significantly more effective in sustaining the E_2_ and AMH levels than the single-injection therapy. These results indicate that hPD-MSC therapy can improve follicular development and concurrently the aging-related decline in E_2_ and AMH hormone production.

The multiple-injection therapy, three injections of hPD-MSCs at 10-day intervals, stimulated early follicular development and steroid biosynthesis in aging ovaries. In contrast, compared to the control group, three injections of hPD-MSCs at 4-week intervals did not significantly affect either the numbers of follicles (Additional file 2: Figure S2a) or the levels of AMH (Additional file 2: Figure S2c). The findings showed that E_2_ levels were significantly reduced 1 week after three injections of hPD-MSCs at 4-week intervals, but thereafter, the E_2_ levels of this group were not significantly different from those of the control group (Additional file 2: Figure S2b). These results suggested that the multiple-injection therapy at 10-day intervals improved ovarian function by follicle development, whereas the multiple-injection therapy at 4-week intervals was not effective. Thus, longer injection intervals for multiple-injection therapy did not show therapeutic effects on follicle development and ovarian hormone production. Because rats have shorter lifespans than humans, 10 rat days is comparable to one human year [29, 30]. The differences in the results between the multiple-injection therapies with short and long intervals in this rat study may provide useful information for designing future clinical tests with older women.

### Changes in circulating miRNAs after hPD-MSC therapy reflect the ovarian reserve and stimulate ovarian function

**Fig. 4:**
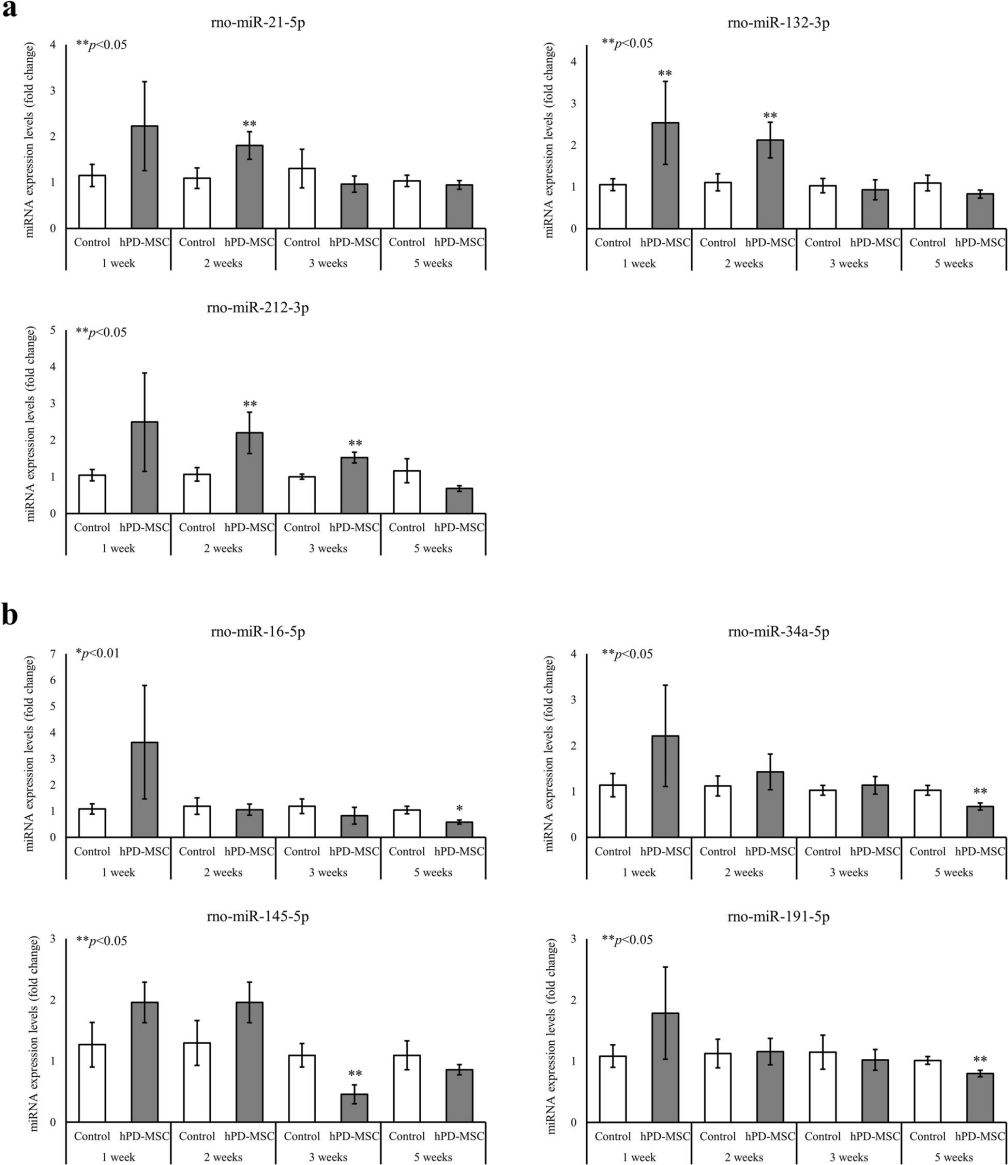
Changes in circulating miRNA levels involved in the ovarian reserve and function after hPD-MSC therapy. **a**, **b** Levels of circulating miRNAs in serum were measured by qRT-PCR. The expression of miR-21-5p, miR-132-3p, and miR-212-3p, related to the ovarian reserve, was significantly increased, whereas the expression of miR-16-5p, miR-34a-5p, miR-145-5p, and miR-191-5p, related to the inhibition of folliculogenesis, was substantially suppressed. The results are presented as the mean ± SEM. Single and double asterisks, * and **, represent statistical significance at *p* < 0.01 and *p* < 0.05, respectively

The altered expression of miRNAs affects folliculogenesis and ovarian steroidogenesis [20, 21, 31]. Because hPD-MSC therapy improved ovarian aging phenotypes**,** we evaluated whether circulating miRNAs could be used as potential biomarkers of the ovarian reserve and ovarian health after hPD-MSC therapy in aged female rats. Seven circulating miRNAs were chosen for further evaluation based on searching the MEDLINE database through PubMed for evidence-based literature associated with ovarian aging with keywords, such as primordial follicle initiation, follicle development, hormone production, and granulosa cell apoptosis [32–37]. The expression of rno-miR16-5p, miR-21-5p, miR-34a-5p, miR-132-3p, miR-145-5p, miR-191-5p, and miR-212-3p in serum was analyzed and normalized to the expression of cel-miR-39. Among the selected circulating miRNAs, miR-21-5p, miR-132-3p, and miR-212-3p were upregulated in the hPD-MSC therapy groups compared with the control group (Fig. 4a), indicating that they could be used to distinguish the aging control groups from the healthier therapy groups. Moreover, the expression of miR-16-5p, miR-34a-5p, miR-145-5p, and miR-191-5p, related to the suppression of primordial follicle initiation and folliculogenesis, was downregulated in the hPD-MSC therapy groups compared with the control group (Fig. 4b).

### Downregulation of miR-145 induces primordial follicle activation by BMPs and the SMAD signaling pathway

**Fig. 5:**
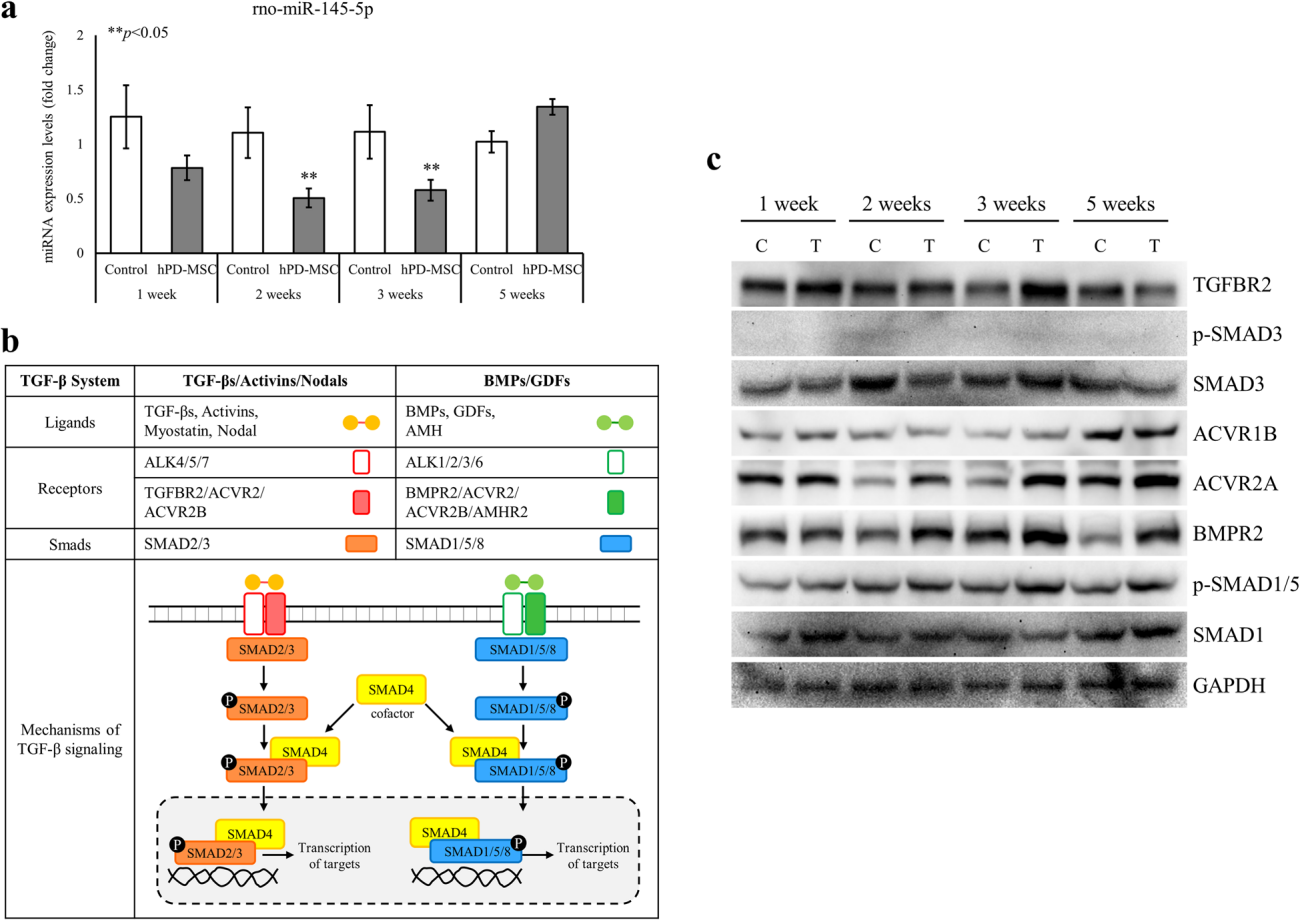
Multiple-injection hPD-MSC therapy-induced miR-145-5p downregulation resulted in increased BMPR2 expression and SMAD pathway activation. **a** Levels of miR-145-5p in serum after multiple-injection therapy were measured by qRT-PCR. The expression of miR-145-5p was significantly reduced after multiple-injection hPD-MSC therapy. The data are presented as the mean ± SEM. The asterisk represents statistical significance at *p* < 0.05. **b** Basic overview of two major SMAD pathways in TGF-β signaling. TGF-βs/Activins/Myostatin/Nodal ligands bind to receptors and lead to the phosphorylation of SMAD2/3, which are translocated into the nucleus and subsequently activate the expression of target genes. However, BMPs/GDFs/AMH ligands activate receptors, phosphorylate SMAD1/5/8, and translocate to the nucleus. In the nucleus, p-SMAD1/5/8 proteins act as transcriptional cofactors that activate target gene expression. **c** Changes in the expression of proteins related to BMP and SMAD signaling in ovaries after the multiple-injection therapy. The expression of BMPR2 and p-SMAD1/5 was significantly increased after multiple-injection hPD-MSC therapy. GAPDH was used as a loading control. C, control group; T, multiple-injection therapy group

We verified that hPD-MSC therapy led to changes in circulating miRNAs involved in the ovarian reserve. We noted that the expression of miR-145-5p was significantly suppressed after single-injection therapy (Fig. 4b). Yang et al. [32] reported that the miR-145 signaling pathway is involved in the initiation of primordial follicle development. Therefore, we examined the expression levels of circulating miR-145-5p after multiple-injection therapy, and as shown in Fig. 5a, the levels of circulating miR-145-5p were markedly decreased in the serum of animals in the cell therapy groups compared with that in the control group at 2 weeks and 3 weeks, similar to the effects of single-injection hPD-MSC therapy.

**Fig. 6:**
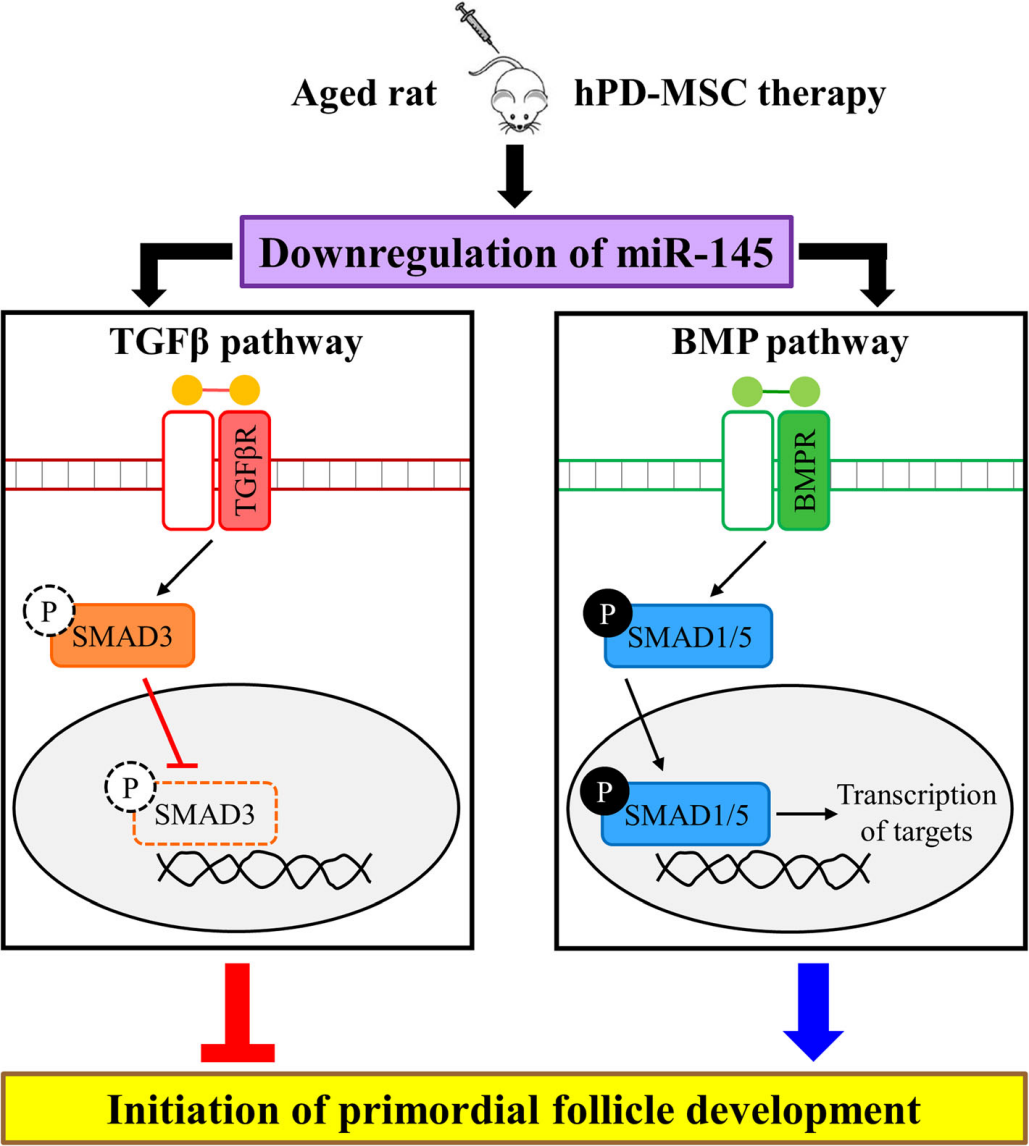
Schematic diagram of the modulation of miR-145 expression and BMP-SMAD signaling by hPD-MSC therapies on folliculogenesis. When old female rats were treated with hPD-MSCs, there was a marked reduction in circulating miR-145 levels and restoration of BMP-SMAD1/5 signaling, but not of TGF-β signaling, resulting in the activation of the primordial-to-primary follicle transition. As a result, aged ovaries had increased ovarian reserve and ovarian function

The transforming growth factor beta (TGF-β) superfamily has been implicated to promote primordial follicle activation [38]. Using numerous computational algorithms, such as TargetScan, miRnada, miRmap, and miRWork, we found that the putative miR-145 targets TGF-β family members and many well-known factors in follicular development, such as *Acvr1b*, *Acvr2a*, *Bmpr2*, *Tgfbr2*, *Smad1*, and *Smad3*. The TGF-β superfamily is broadly divided into two major classes: (1) TGF-βs, Activins, Myostatin, and Nodal, which act via SMAD2 and SMAD3, and (2) BMPs, GDFs, and AMH, which act via SMAD1, SMAD5, and SMAD8 [39]. TGF-β family ligands bind to receptor serine kinases that phosphorylate SMAD proteins, which form transcriptional complexes that regulate specific genes (Fig. 5b). Thus, we analyzed whether the downregulation of circulating miR-145-5p caused by multiple-injection therapy affected the expression of these putative target proteins in ovaries identified by computational algorithms. As shown in Fig. 5c, the expression level of the TGFBR2 protein was relatively high after multiple-injection therapy. However, the expression of p-SMAD3, which is downstream of TGF-β, was not detected in ovaries with or without hPD-MSC stimulation, indicating that TGF-β signaling is not involved in primordial follicle activation (Figs. 5c and 6). The protein expression of ACVR2A was markedly upregulated, whereas no change in ACVR1B protein expression was observed in the multiple-injection hPD-MSC therapy group compared with the control group (Fig. 5c). The BMP receptor and SMAD1/5/8 are downstream targets of BMP signaling in a positive feedback loop. Enhanced BMPR2 protein expression was observed in ovaries 2–5 weeks after multiple-injection hPD-MSC therapy (Fig. 5c). Likewise, p-SMAD1/5 expression was significantly increased in ovaries after multiple-injection hPD-MSC therapy (Fig. 5c). These findings strongly suggest that hPD-MSC therapy promotes the primordial-to-primary follicle transition through stem cell therapy-derived BMP signaling (Fig. 6).

## Discussion

In the current study, the effects of single- and multiple-injection hPD-MSC therapy on ovarian aging were explored. hPD-MSCs were found in the ovaries after multiple-injection therapy and single-injection therapy, as we expected, and we observed attenuated ovarian dysfunction and increased ovarian reserve in old rat ovaries after hPD-MSC therapy. Importantly, our results showed that hPD-MSC transplantation through multiple injections dramatically increased the number of primary follicles through the primordial-to-primary follicle transition in aged ovaries, rescued hormone levels, and improved ovarian functions. The multiple-injection therapy changed the expression of circulating miRNAs and proteins associated with follicle development via BMP signaling and steroidogenesis in ovaries. In our experiments, compared to the single-injection therapy, the multiple-injection therapy was more effective in ovarian stimulation, increasing ovarian reserve, and functions, which suggested that multiple transplantations might be more valuable to clinically treat patients with ovarian dysfunction caused by aging.

Recently, because rodents are very useful models to study follicular development and ovarian function, it is important to know the similarities and differences in ovarian aging between rodents and humans. Previous work has established that SD rats at approximately 8–10 months of age undergo the reproductive aging process, showing decreased numbers of developing follicles and altered estrous cyclicity [40, 41]. In SD rats, a decline in fertility continues gradually until 12 months (approximately 52 weeks) of age [41]. From 12 months old onward, SD rats are infertile due to the absence of successful pregnancies and pups born per rat [42]. Therefore, for functional analysis after hPD-MSC therapy in ovaries with reproductive senescence, we used 12-month-old (52–54 weeks) female SD rats.

Menopause caused by aging and/or chemotherapy results in impaired hormone biosynthesis that causes metabolic syndrome, heart disease, depression, dementia, osteoporosis, atrophic vaginitis, and sexual dysfunction [4]. Recently, hormone replacement therapy has been the most effective treatment available for the disruptive symptoms of ovarian aging, which carries a risk for breast cancer and other diseases, including thromboembolism, stroke, vaginal bleeding, and heart disease [43, 44]. Previous studies established that mesenchymal stem cells (MSCs) can be used to treat animal models with chemotherapy- or OVX-induced early menopause as they ameliorate ovarian function by stimulating folliculogenesis [25, 45–47]. In this study, our results showed that the number of developing follicles and levels of hormones were significantly increased after hPD-MSC therapy in aged rats. After single- or multiple-injection therapy, hPD-MSCs did not induce xenogeneic immune responses in aged rats, indicating the low immunogenicity of hPD-MSCs. These results suggest that hPD-MSCs may represent an important cell source with clinical applications for improving ovarian function in older women.

The altered expression of miRNAs may affect folliculogenesis and ovarian steroidogenesis. Since the discovery that circulating miRNAs can be detected in different biofluids, including serum and follicular fluid, it has become evident that miRNAs can be used as novel biomarkers for the clinical diagnosis of ovarian health [20, 21, 48]. When rat ovaries with chemotherapy-induced damage were injected with stem cells stably expressing miR-21, improvements in ovarian structure and functions via the inhibition of granulosa cell apoptosis were observed [33]. Moreover, miR-132 upregulation in mouse granulosa cells promotes the production of E_2_ by the translational repression of Nurr1 [34]. In contrast, low expression of miR-16 and miR-191 in buffaloes is involved in ovarian follicular dominance and prevents ovarian follicular atresia [35]. In addition, downregulation of miR-34a and miR-145 resulted in suppression of granulosa cell apoptosis in a porcine model and activation of primordial follicle development in mice, respectively [32, 36]. Importantly, we showed that the levels of miR-21 and miR-132 were increased, whereas the expression of miR-16, miR-34a, miR-145, and miR-191 was decreased after hPD-MSC therapy. These data indicate that hPD-MSC therapy leads to the altered expression of miRNAs related to folliculogenesis, hormone synthesis, granulosa cell apoptosis, and follicle atresia. Further studies examining the finely tuned miRNA-mediated regulation of ovarian function with aging after hPD-MSC therapy are needed.

The TGF-β superfamily consists of secreted multifunctional proteins including TGF-βs, AMH, activins, BMPs, and GDFs. These proteins are developmentally important growth factors that function via paracrine or autocrine signaling [49]. Currently, the TGF-β superfamily is divided into two groups: TGF-βs/activins and BMPs/GDFs [39]. TGF-β superfamily ligands bind to serine/threonine kinase receptor type I (activin-like kinases; ALKs) and II (ACVR2A, ACVR2B, BMPR2, AMHR2, and TGFBR2) complexes on the cell surface that activate the SMAD pathway through the phosphorylation of SMAD proteins. Specifically, TGF-βs, Activins, and Nodals are mediated by SMAD2 and SMAD3, while BMPs, GDFs, and AMH are mediated by SMAD1, SMAD5, and SMAD9. The phosphorylated SMAD proteins associate with SMAD4, translocate from the cytoplasm to the nucleus, and then regulate target gene transcription [50, 51]. In the ovary, TGF-β superfamily members are expressed in the oocyte, granulosa cells, and thecal cells at different follicle stages and contribute to the regulation of folliculogenesis [52]. In early folliculogenesis, TGF-βs play pivotal roles in the maintenance of primordial follicle pools and in the activation of primordial follicles via SMAD-dependent and/or SMAD-independent pathways [53, 54]. BMPs have also been implicated as positive intraovarian regulators of the primordial-to-primary follicle transition [38]. BMPR2 is expressed in the granulosa cells of primordial follicles in ruminants and in preantral follicles in rodents [55], which is in accordance with the high protein expression levels of BMPR2 in the ovaries after multiple-injection therapy in this study. In the present study, our results showed that SMAD1 and p-SMAD1/5 were highly expressed in ovaries after multiple-injection hPD-MSC therapy, suggesting that multiple-injection hPD-MSC therapy activated the classic BMP pathway characterized by SMAD1/5/8 signaling and activated the initiation of primordial follicle development in aged ovaries. Collectively, these results confirm the importance of hPD-MSC therapy-induced BMP-SMAD signaling in the primordial-to-primary follicle transition, which is critical for the ability to treat and/or attenuate female ovarian aging.

## Conclusions

We demonstrated that the activation of the intraovarian BMP-SMAD1/5 signaling pathway by the downregulation of circulating miR-145 with stem cell therapy is essential for coordinating key events of the primordial-to-primary follicle transition in ovaries at advanced age (Fig. 6). Our results provide evidence that women with ovarian dysfunction accompanied by aging may have the option to use hPD-MSCs to rejuvenate their ovaries and to attenuate the effects of menopause via the return of ovarian function.

## Supplementary Information

**Additional file 1 : Figure S1.** Analysis of human cells after a single injection of hPD-MSCs into aged rats via the tail vein**.** Human DNA (*AluYb8*) sequences were found in different organs (lung, liver, and ovary) after single-injection hPD-MSC therapy. The results show that the hPD-MSCs were located in the ovary 2–3 weeks after the tail vein injection. Red boxes indicate the present of the *AluYb8* sequence in organs.

**Additional file 2 : Figure S2.** Three rounds of hPD-MSC therapy at longer injection intervals had no therapeutic effects on ovarian aging. (**a**) After three injections at 4-week intervals, the number of follicles at various stages was counted and compared with the number in the control group. At the indicated time-points following the hPD-MSC injection, six rats from each group were randomly selected and analyzed after H&E staining in every tenth section throughout the ovary. The results are presented as the mean ± SEM. (**b**, **c**) Serum levels of E_2_ (**b**) and AMH (**c**) as determined by ELISA at various time-points after three injections at 4-week intervals showed no difference. Data are presented as the mean ± SEM. The asterisk represents statistical significance at *p* < 0.05.

## Data Availability

All data are included in the text and supplementary information.

## Abbreviations

AMH: Anti-Müllerian hormone
BMP: Bone morphogenetic protein
E_2_: Estradiol
hPD-MSCs: Human placenta-derived mesenchymal stem cells
Ovx: Ovariectomy
TGF-β: Transforming growth factor β
