# Supplementary material for: Human placenta-derived mesenchymal stem cells stimulate ovarian function via miR-145 and bone morphogenetic protein signaling in aged rats
Source: Stem Cell Res Ther. 2020 Nov 5;11:472. doi: 10.1186/s13287-020-01988-x (PMC7643421; doi:10.1186/s13287-020-01988-x)
Supplement: Supplementary file 1 — Additional file 1 : Figure S1. Analysis of human cells after a single injection of hPD-MSCs into aged rats via the tail vein. Human DNA (AluYb8) sequences were found in different organs (lung, liver, and ovary) after single-injection hPD-MSC therapy. The results show that the hPD-MSCs were located in the ovary 2–3 weeks after the tail vein injection. Red boxes indicate the present of the AluYb8 sequence in organs. [file 13287_2020_1988_MOESM1_ESM.docx]

**
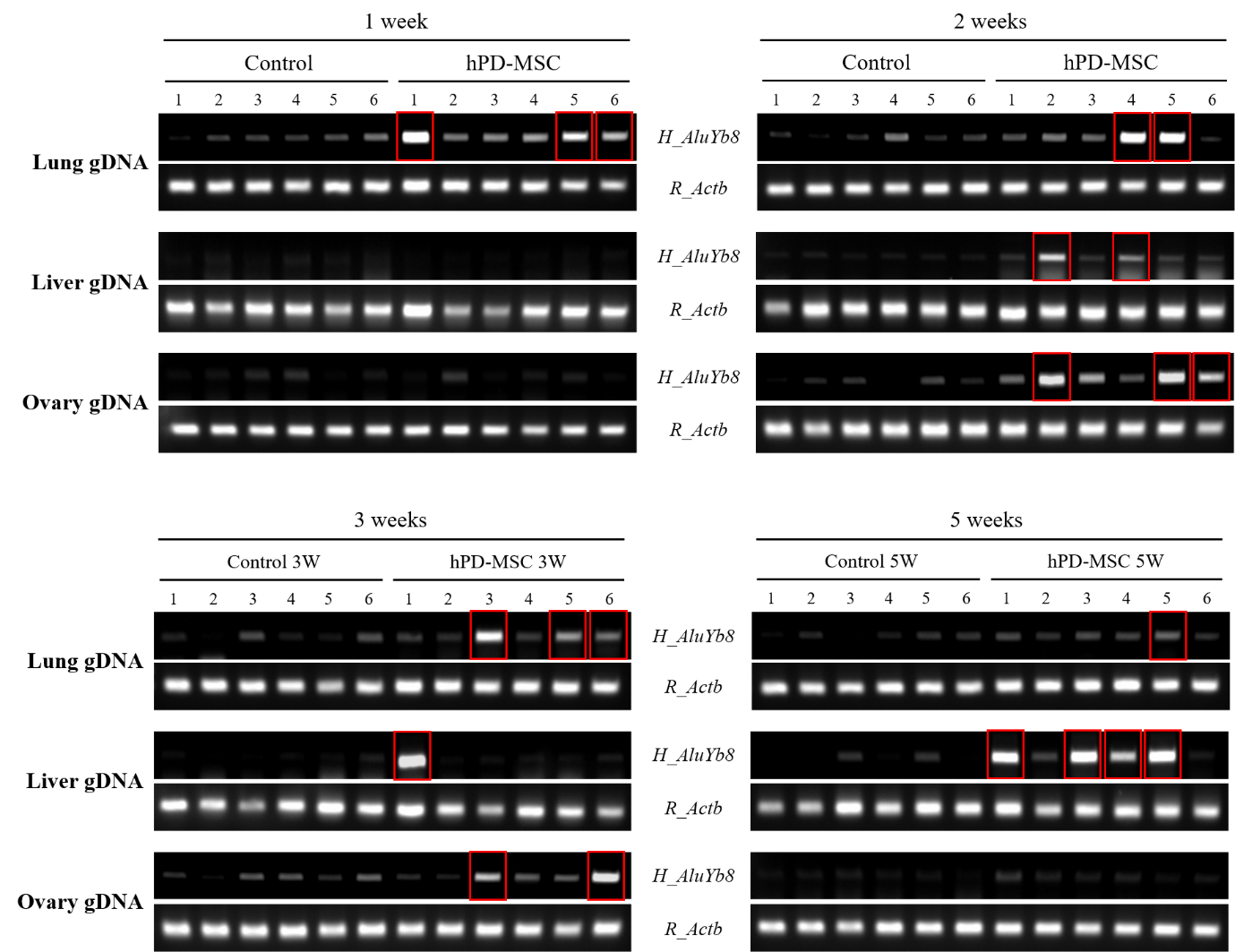
**

**Figure S1.** Analysis of human cells after a single injection of hPD-MSCs into aged rats via the tail vein. Human DNA (*AluYb8*) sequences were found in different organs (lung, liver and ovary) after single-injection hPD-MSC therapy. The results show that the hPD-MSCs were located in the ovary 2-3 weeks after the tail vein injection. Red boxes indicate the present of the *AluYb8* sequence in organs.
