# Supplementary material for: Human placenta-derived mesenchymal stem cells stimulate ovarian function via miR-145 and bone morphogenetic protein signaling in aged rats
Source: Stem Cell Res Ther. 2020 Nov 5;11:472. doi: 10.1186/s13287-020-01988-x (PMC7643421; doi:10.1186/s13287-020-01988-x)
Supplement: Supplementary file 2 — Additional file 2 : Figure S2. Three rounds of hPD-MSC therapy at longer injection intervals had no therapeutic effects on ovarian aging. (a) After three injections at 4-week intervals, the number of follicles at various stages was counted and compared with the number in the control group. At the indicated time-points following the hPD-MSC injection, six rats from each group were randomly selected and analyzed after H&E staining in every tenth section throughout the ovary. The results are presented as the mean ± SEM. (b, c) Serum levels of E2 (b) and AMH (c) as determined by ELISA at various time-points after three injections at 4-week intervals showed no difference. Data are presented as the mean ± SEM. The asterisk represents statistical significance at p < 0.05. [file 13287_2020_1988_MOESM2_ESM.docx]

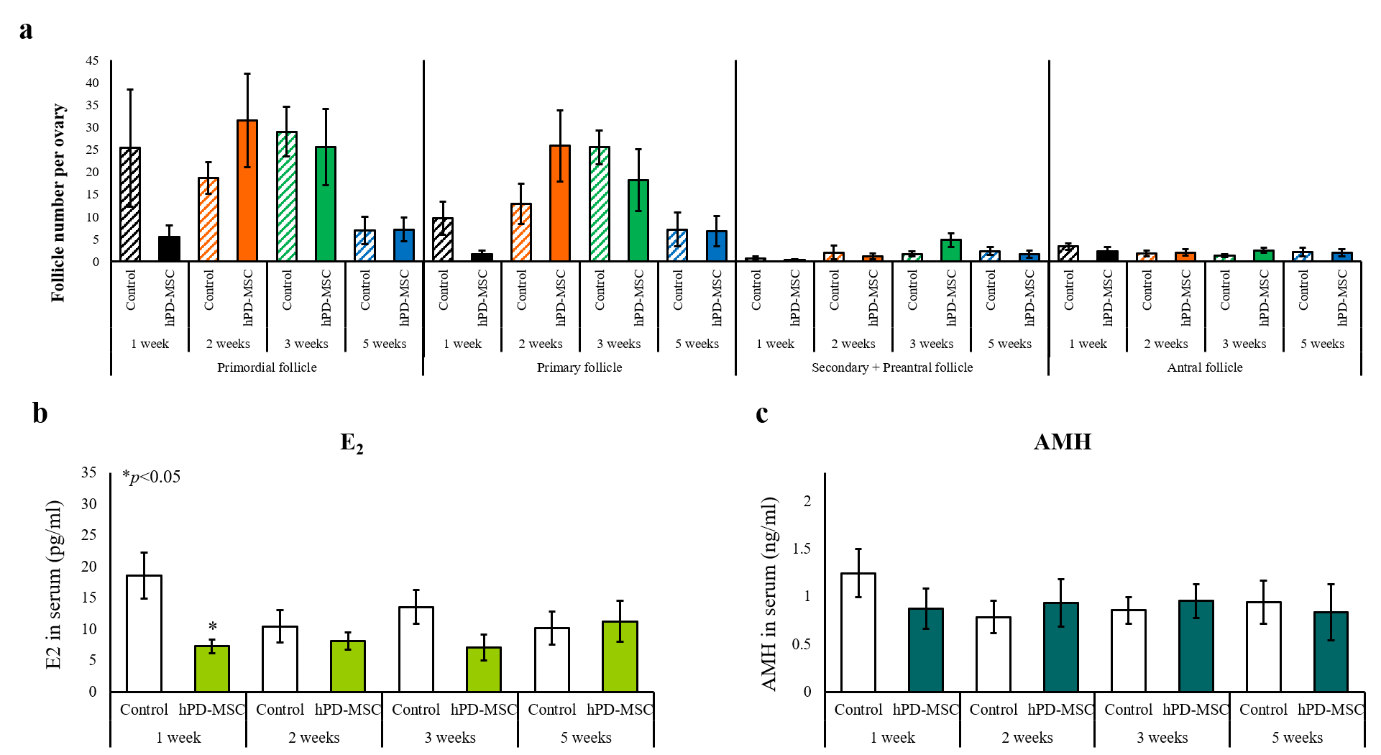


**Figure S2.** Three rounds of hPD-MSC therapy at longer injection intervals had no therapeutic effects on ovarian aging. (a) After three injections at 4-week intervals, the number of follicles at various stages was counted and compared with the number in the control group. At the indicated timepoints following hPD-MSC injection, six rats from each group were randomly selected and analyzed after H&E staining in every tenth section throughout the ovary. The results are presented as the mean ± SEM. (b-c) Serum levels of E_2_ (b) and AMH (c) as determined by ELISA at various timepoints after three injections at 4-week intervals showed no difference. Data are presented as the mean ± SEM. The asterisk represents statistical significance at *p*<0.05.
